# The Impact of Different Velocity Losses on Post-Activation Performance Enhancement (PAPE) Effects in Sprint Athletes: A Pilot Randomized Controlled Study

**DOI:** 10.3390/sports12060157

**Published:** 2024-06-06

**Authors:** Liang Li, Ling Mo, Yanxu Liu, Tao Mei

**Affiliations:** 1 China Institute of Sport and Health Science, Beijing Sport University, Beijing 100084, China; p20212002968@siswa.upsi.edu.my (L.L.); bsumoling@163.com (L.M.); 15846688922@163.com (Y.L.); 2 Faculty of Sport Science and Coaching, Sultan Idris Education University, Tanjung Malin 35900, Malaysia

**Keywords:** post-activation performance enhancement, velocity-based strength training, velocity loss, sprinter

## Abstract

Post-activation performance enhancement (PAPE) can significantly improve athletic performance. This study investigated the effects of two different velocity loss (10% VL and 20% VL) protocols on PAPE in 20 m sprint performance among sprint athletes. Twenty-four male sprint athletes (100 m sprint time: 10.96 ± 0.15 s) participated in the study. A randomized crossover experimental design was used to compare the traditional group (TG) and 10% VL and 20% VL interventions. Sprint tests were conducted at 4, 8, 12, and 16 min post-intervention. A two-way repeated measures ANOVA revealed a significant interaction effect between group and time on 20 m sprint performance (F = 2.817, *p* = 0.037, partial η^2^ = 0.585). Simple main effects analysis revealed significant improvements at 4 min for the 20% VL group (*p* < 0.05). Cohen’s d values indicated improvements in 10 m sprint times at 8 min for all groups (TG: effect size (ES) = −0.270, 10% VL: ES = −0.038, 20% VL: ES = −0.279). Improvements in 20 m sprint times were observed at 4 min for the 20% VL group (ES = −0.296) and at 16 min for the 10% VL group (ES = −0.276). In conclusion, the velocity loss-based PAPE protocol (20% VL) demonstrated a superior induction of PAPE effects in sprint athletes at 4 min compared to traditional 1RM-based PAPE protocols. However, no significant differences were observed between the two protocols at 8, 12, and 16 min.

## 1. Introduction

Pre-event warm-up activities are crucial for enhancing athletes’ performance, as they can elevate the body temperature, increase the speed of neural impulse conduction, and alleviate pre-competition anxiety [1]. Post-activation performance enhancement (PAPE) refers to a physiological phenomenon where brief high-intensity resistance training conducted beforehand leads to an increase in muscle explosiveness and force generation speed [2]. Unlike the classic post-activation potentiation (PAP) effect, the enhancement effect of PAPE on muscle strength can last for several minutes after the activity, whereas the effect of PAP reaches its maximum shortly after the activity, approximately around 28 s [3]. PAP is typically defined as the phenomenon where muscle strength performance is instantly enhanced following high-intensity muscle contraction activities [4]. This phenomenon is attributed to central nervous system activation and muscle enhancement effects elicited by prior high-intensity muscle contraction activities, resulting in an immediate increase in muscle contraction force and power output. Unlike PAP, the mechanism of PAPE may involve factors such as muscle temperature, muscle/cellular water content, and muscle activation [3,5]. In terms of evaluation methods, PAP is typically assessed by measuring immediate changes in muscle strength or power output—for example, using force platforms for vertical jump tests or squat tests to assess the immediate enhancement effect on strength. The evaluation of PAPE focuses more on longer-term effects, and may include repeated testing of specific athletic skills (such as vertical jumps, sprints, etc.) and comparing performance differences between tests conducted after and before high-intensity activities.

PAPE has been applied across various sports disciplines to enhance athletic performance. Interventions involving squats, with either a 90% 1RM for three repetitions or 60% 1RM for six repetitions, can lead to a slight increase in mean power output for sprinters, although there is no statistically significant difference between the two different loads. In pre-event warm-up activities for sprinters, using a 10% load can be more effective in improving the 20 m sprint performance of elite female sprinters, compared to conditioning activities incorporating resistance training with 5% or 15% of body mass [6]. Additionally, squat conditioning activities with elastic bands can also improve sprinters’ performance [7].

In the current formulation of protocols inducing PAPE effects, most studies have used conditioning activities based on the maximum number of repetitions (Repetition Maximum, RM) to determine the intensity of the load. However, these conditioning activities have the problem of being unable to accurately control intensity [8,9]. Such loading conditioning activities (e.g., 90% 1RM squats) may overlook individual differences in the physical function status of different athletes. The fundamental issue with these differences is that the selected load intensity (such as RM) cannot accurately reflect the neuromuscular status of the athletes. Factors such as athletes’ nutrition, sleep, and fatigue status can affect their neuromuscular status and athletic performance [10]. An athlete’s performance can fluctuate daily due to variations in readiness, including fatigue, recovery level, and mental state, which can significantly impact performance. Effective preparation and recovery strategies are crucial to ensuring that athletes achieve their best performance on game day [11]. Therefore, to better design loading conditioning activities for PAPE, it is necessary to further optimize these conditioning activities.

In strength training, as muscle fatigue accumulates, the execution speed of movements may decrease. Utilizing movement speed to adjust or formulate training loads is a potential approach to address the issue of precise load monitoring [10]. Velocity loss (VL), which refers to the percentage decrease in movement speed relative to the initial speed, is a commonly used indicator to quantify movement speed. Studies have found a high correlation between 1RM and movement speed in different strength-training exercises (r = 0.998) [12]. Research analyzing the load–velocity relationship in exercises such as push-ups, pull-ups, half squats, full squats, and leg presses has also revealed a strong correlation between load magnitude and bar speed (predictive capacity equation R^2^ = 0.96–0.98), which is independent of training background and athletes’ strength levels [13]. Therefore, in addressing the issue of objectively quantifying and monitoring athletes’ actual training loads, it is suggested to monitor the load based on the magnitude of VL achieved in each set of strength training, rather than being limited to fixed repetitions prescribed by relative loads (%1RM). Additionally, monitoring the VL load can not only ensure the accurate matching of target intensity with actual training intensity but also control the level of fatigue during each set, avoiding excessive fatigue and achieving consistency in stimulus levels among individuals [14,15].

Regarding the selection range of VL, studies have found that 20% VL is a critical value, where a VL lower than 20% is more conducive to rapid strength improvement, while a VL exceeding 20% is more conducive to muscle hypertrophy (20–40% VL). When the difference in VL is within 10%, there seems to be no significant difference in training effects [16]. However, there is considerable controversy surrounding the critical value of 20% VL in many studies. Some research suggests that, compared to 20% VL, strength training with 10% VL is more beneficial for explosiveness and muscle strength gains [17]. Meanwhile, studies have also compared the relationship between 10% VL and 20% VL with a 15 m sprint time and found that both VLs achieve similar effects [18]. So far, there is no evidence to suggest that the effect of velocity loss-induced PAPE is necessarily superior to traditional load-based RM conditioning activities, and this requires further exploration. Additionally, stride length and stride frequency are fundamental components of sprinting biomechanics, directly impacting athletes’ speed and efficiency [19,20]. Understanding the complex relationship between training interventions, biomechanical parameters such as stride length and frequency, and PAPE outcomes can provide valuable insights for tailoring more effective PAPE protocol designs for sprint athletes.

Therefore, this study, with elite sprinters as participants, sets two velocity loss load intensities of 20% VL and 10% VL as the experimental groups, and a traditional %1RM load intensity as the control group, to investigate whether different velocity loss conditioning activities may cause PAPE phenomena in 20 m sprint performance in sprinters. The goal is to provide new insights into the personalized load adjustment of PAPE training programs in the daily training and pre-competition preparation activities of sprinters. It was hypothesized that (1) training programs based on velocity loss (VL) load intensity and traditional %1RM load intensity can both improve sprint performance and (2) stride length and stride frequency are potential factors influencing sprint performance.

## 2. Materials and Methods

### 2.1. Participants

The participant inclusion criteria were as follows: ① Male participants. ② At least one year of resistance training experience, including familiarity with squat exercises. ③ Participants must have a squat 1RM value ≥ 1.5 times their body mass, confirmed through a 1RM test. ④ Good health, with no joint injuries (open or closed) or cardiovascular diseases that would render them unsuitable for exercise intervention. ⑤ Classified as level 2 athletes or higher. Exclusion criteria included an inability to complete the intervention or incomplete data. Twenty-four male sprinters voluntarily participated in this study (mean age: 20.19 ± 1.72 years, height: 179.90 ± 4.96 cm, body mass: 70.41 ± 4.78 kg, squat 1RM: 138.53 ± 14.24 kg, 100 m sprint time: 10.96 ± 0.15 s). This study was approved by the Ethics Committee of Sports Science at Beijing Sport University. All participants received full information regarding the purpose and procedures of the experiment before participating and provided written informed consent. This study has been registered with the Chinese Clinical Trial Registry (ChiCTR) under registration number ChiCTR2400084283. The trial adhered to ethical guidelines to ensure compliance with ethical standards and protect the rights of participants.

### 2.2. Training Protocol

The study utilized a randomized crossover experimental design, where participants were randomly assigned intervention orders by drawing lots. Participants allocated to the traditional group (TG) underwent the traditional training protocol based on 1RM load for their intervention initially, followed by crossover to either the 10% VL group or the 20% VL group after a washout period and so forth. The allocation ratio for each intervention was 1:1:1, and participants completed all three interventions. Detailed instructions and training were provided to all participants before the experiment, including familiarization with the intervention protocols (squat training and velocity-based training), to ensure they understood and could correctly perform the training interventions. Emphasis was placed on maximal velocity intent for each repetition, encouraging participants to achieve the training targets as effectively as possible.

In the TG group: (1) Participants underwent a standardized warm-up lasting 30–40 min before the formal intervention. (2) The first 20 m sprint test was conducted. (3) A 5 min rest followed. (4) Participants performed 3 squats at 85% of their 1RM to familiarize themselves with the movement (the weight being determined based on the participant’s 1RM test prior to enrollment). (5) A 4 min rest followed. (6) Participants engaged in squat training at 85% of their 1RM weight, completing 6 repetitions per set for 2 sets with a 2 min rest interval between sets. (7) Sprint times were recorded using a Smartspeed timer at 4, 8, 12, and 16 min after completing the squats. Additionally, an Optojump system (OptoJumpNext, Microgate, Bolzano, Italy) collected information on stride length and frequency.

In the 10% VL Group: (1) Participants underwent a standardized warm-up lasting 30–40 min before the formal intervention. (2) The first 20 m sprint test was conducted. (3) A 5 min rest followed. (4) Participants performed 3 squats at 85% of their 1RM to familiarize themselves with the movement (the weight being determined based on the participant’s 1RM test prior to enrollment), with a Gymware linear sensor recording the highest average speed of the 3 squats. (5) A 4 min rest followed. (6) Participants engaged in squat training at 85% of their 1RM weight, aiming for a 10% velocity loss (10% VL) at the end of the squat training. The training consisted of 2 sets, with a 2 min rest interval between sets. (7) Sprint times were recorded using the Smartspeed timer at 4, 8, 12, and 16 min after completing the squats. Additionally, the Optojump evaluation system collected information on stride length and frequency.

**Figure 1:**
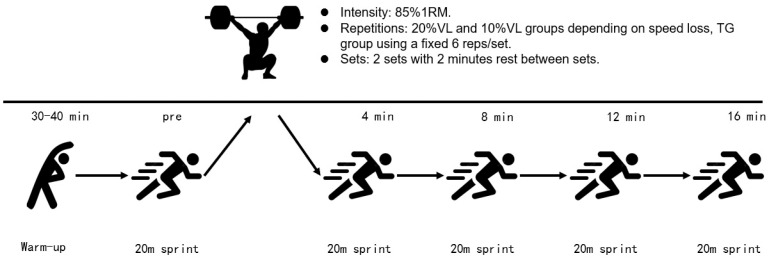
The intervention for participants.

In the 20% VL Group: The intervention mirrored that of the 10% VL group, except that participants aimed for a 20% velocity loss in step (6). The experimental procedure is depicted in Figure 1.

### 2.3. Measurement

#### 2.3.1. Movement Speed Monitoring

The Gymaware linear sensor (Gymaware RS, Kinetic Performance Technology, Canberra, Australia) was employed to monitor the vertical movement speed of the barbell during the participants’ squat training. The repetition count was determined based on the VL target (10% VL group and 20% VL group). Prior to squatting, the Gymaware device was positioned on one side of the squat rack, and the participant’s weight and 85% 1RM weight were inputted into the computer. Participants were instructed to squat with a load corresponding to 85% 1RM, and the squatting was stopped when the participant reached 10% VL or 20% VL. Each participant underwent three tests. The Gymaware software (Version 4.2.0, Kinetic Performance Technology, Canberra, Australia) recorded the participants’ movement speed in real time, capturing the highest average speed during the three squatting sessions.

#### 2.3.2. 20 m Sprint Test

The Smartspeed segmented timing real-time feedback system (SmartSpeed Pro V2.1, VALD Performance, Queensland, Australia) was utilized to collect the time taken by the participants for the 10 m and 20 m sprints. During the test, participants adopted a fixed spike shoe and a crouching start position. To ensure the accuracy of the test, participants recorded the distance between their feet and the angle of the force plate under their feet before each test. To prevent premature contact with the infrared sensor, the support points for the participants’ hands were set at a certain distance behind the instrument. At the start of the test, participants indicated their readiness to the tester, and the experimenter manually clicked the start button upon hearing the “beep” sound. Participants then commenced the sprint. As the participants passed each segment of infrared emitted by the transmitter to the receiver, the sprint data collection was completed.

#### 2.3.3. Stride Length and Frequency Testing

The Optojump system (OptoJumpNext, Microgate, Bolzano, Italy) was utilized to collect the stride length and frequency indicators during the participants’ sprints. The validity and reliability of this instrument have been confirmed in previous studies [21]. The Optojump system has a testing range of 20 m. Prior to the testing, the Optojump transmitter and receiver units were placed parallel and flat on both sides of the digital running track, and the connectors were then inserted sequentially. The Optojump software (Version 1.13.17, Microgate, Bolzano, Italy) was opened on the computer, and basic information about the participants (name, age, height, body mass, etc.) was inputted. The repeated sprint test item was selected, and the test was initiated by clicking “execute”, indicating to the participants to begin the test. Data collection commenced when either foot of the participant touched the ground, and the infrared in the transmitter and receiver units was successfully detected. The data were saved after the participant exited the Optojump detection area.

### 2.4. Statistical Analysis

SPSS 27.0 (IBM SPSS, Chicago, IL, USA) was utilized as the statistical software for data analysis. The descriptive statistics were presented in the form of “mean ± standard deviation” (M ± SD). The normality of data within each group was assessed using the Shapiro–Wilk test. A two-way repeated measures analysis of variance (group × time) was conducted for the research data, with the between-group factor being the grouping factor (TG group, 10% VL group, 20% VL group) and the within-group factor being the time factor (pre, 4 min, 8 min, 12 min, 16 min). In the presence of a significant interaction effect, a simple main effects analysis was performed for each factor (with a significance level set at *p* < 0.05). For the group factor and the time factor, we conducted Tukey’s honestly significant difference (HSD) test to control the error rate in multiple comparisons. Cohen’s d value was computed as the effect size measure to evaluate the differences within the groups, using an absolute value direction scale. The evaluation criteria were as follows: 0 < |ES| < 0.2 for a very small effect size; 0.2 < |ES| < 0.5 for a small effect size; 0.5 < |ES| < 0.8 for a moderate effect size; |ES| > 0.8 for a large effect size.

## 3. Results

### 3.1. Effects of Different Velocity Losses on PAPE in 0–10 m Phase

Following the intervention, a repeated measures analysis of variance revealed no significant interaction between the time effect and group intervention effect for the 0–10 m sprint performance (F = 2.223, *p* = 0.083, partial η^2^ = 0.526). A time main effect analysis indicated that the sprint times for the 0–10 m phase at each testing point (4 min, 8 min, 12 min, 16 min) did not significantly decrease across the three groups (F = 1.474, *p* = 0.247, partial η^2^ = 0.228). The main effect of group intervention showed no statistically significant differences in the sprint times for the 0–10 m phase among the three groups at each testing point (F = 1.931, *p* = 0.169, partial η^2^ = 0.149) (Table 1).

**Figure 2:**
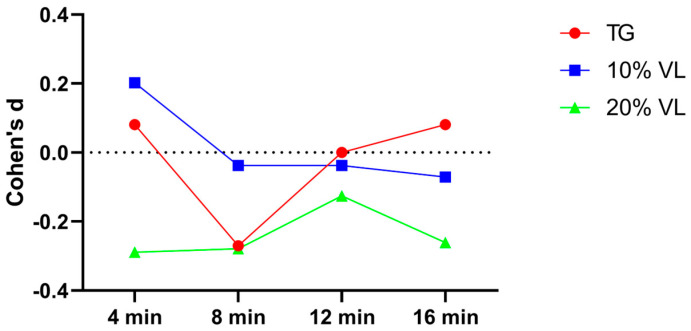
Cohen’s d value of TG, 10% VL, and 20% VL interventions in 0–10 m phase.

A subsequent effect size analysis revealed that the effect sizes for the TG group at each testing point were 0.081, −0.270, 0, and 0.081, respectively. For the 10% VL group, the effect sizes at each testing point were 0.202, −0.038, −0.038, and −0.071, respectively. As for the 20% VL group, the effect sizes at each testing point were −0.289, −0.279, −0.126, and −0.261, respectively (Figure 2).

### 3.2. Effects of Different Velocity Losses on PAPE in 10−20 m Phase

Following the intervention, a repeated measures analysis of variance revealed no significant interaction between the time effect and group intervention effect for the 10–20 m sprint performance (F = 0.969, *p* = 0.493, partial η^2^ = 0.326). A time main effect analysis indicated that the sprint times for the 10–20 m phase at each testing point (4 min, 8 min, 12 min, 16 min) did not significantly decrease across the three groups (F = 1.539, *p* = 0.237, partial η^2^ = 0.123). The main effect of group intervention showed no statistically significant differences in the sprint times for the 10–20 m phase among the three groups at each testing point (F = 0.903, *p* = 0.481, partial η^2^ = 0.153) (Table 2).

**Figure 3:**
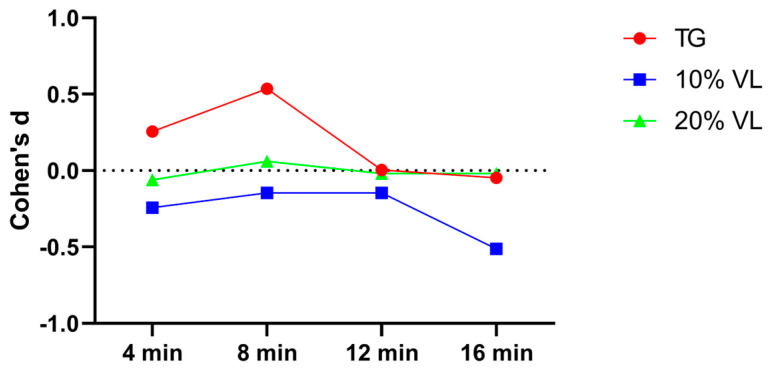
Cohen’s d value of TG, 10% VL, and 20% VL interventions in 10–20 m phase.

A subsequent effect size analysis revealed that the effect sizes for the TG group at each testing point were −0.204, −0.056, −0.241, and −0.167, respectively. For the 10% VL group, the effect sizes at each testing point were 0.067, 0, −0.107, and 0.053, respectively. As for the 20%VL group, the effect sizes at each testing point were −0.079, 0.095, 0.032, and 0.048, respectively (Figure 3).

### 3.3. Effects of Different Velocity Losses on PAPE in 0–20 m Phase

Following the intervention, a repeated measures analysis of variance revealed a significant interaction between the time effect and group intervention effect for the 0–20 m sprint performance (F = 2.817, *p* = 0.037, partial η^2^ = 0.585). Further analysis indicated that the 20% VL group exhibited significant differences at 4 min (*p* < 0.05). Regarding the main effect of time, participants across all three groups did not show significant decreases in sprint times for the 0–20 m phase at each testing point (F = 0.852, *p* = 0.509, partial η^2^ = 1.146). Similarly, there were no statistically significant differences in sprint times for the 0–20 m phase among the three groups at each testing point for the main effect of group intervention (F = 1.687, *p* = 0.208, partial η^2^ = 0.133) (Table 3).

**Figure 4:**
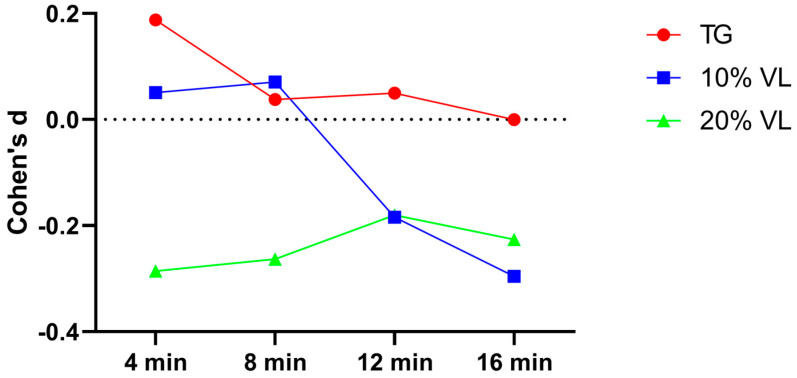
Cohen’s d value of TG, 10% VL, and 20% VL interventions in the 0–20 m phase.

The calculated Cohen’s d values for individual effect sizes were as follows: For the TG group, the effect sizes at each testing point were 0.188, 0.038, 0.05, and 0. For the 10% VL group, the effect sizes were 0.051, 0.071, −0.184, and −0.296, respectively. As for the 20% VL group, the effect sizes were −0.286, −0.263, −0.180, and −0.226, respectively (Figure 4).

### 3.4. Impact of Different Velocity Losses on Stride Length in 0–20 m Phase

The results of the repeated measures analysis of variance indicated that there was no significant interaction between the time effect and group intervention effect for stride length in the 0–20 m phase (F = 1.065, *p* = 0.388, partial η^2^ = 0.034). Regarding the main effect of time, participants across all three groups did not exhibit significant changes in stride length for the 0–20 m phase at each time point (F = 2.282, *p* = 0.061, partial η^2^ = 0.036). Similarly, for the main effect of group intervention, there were no significant differences in stride length among the different intervention groups at each time point (F = 1.856, *p* = 0.159, partial η^2^ = 0.015) (Table 4).

**Figure 5:**
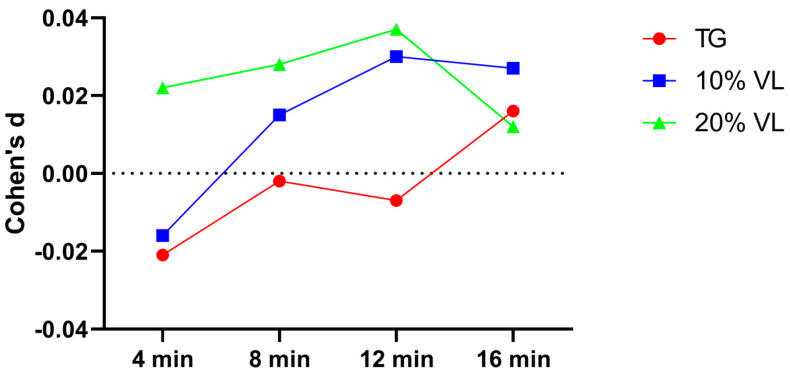
Stride length Cohen’s d value of TG, 10% VL, and 20% VL interventions in 0–20 m Phase.

Individual effect sizes calculated using Cohen’s d values revealed that for the TG group, the effect sizes at each testing point were −0.021, −0.002, −0.007, and 0.016, respectively. For the 10% VL group, the effect sizes were −0.016, 0.015, 0.030, and 0.027, respectively. As for the 20% VL group, the effect sizes were 0.022, 0.028, 0.037, and 0.012, respectively (Figure 5).

### 3.5. Impact of Different Velocity Losses on Stride Frequency in 0–20 m Phase

The results of the repeated measures analysis of variance revealed no significant interaction between the time effect and group intervention effect for stride frequency in the 0–20 m phase (F = 0.392, *p* = 0.924, partial η^2^ = 0.013). Regarding the main effect of time, participants across all three groups did not exhibit significant changes in stride frequency for the 0–20 m phase at each time point (F = 0.625, *p* = 0.645, partial η^2^ = 0.010). Similarly, for the main effect of group intervention, there were no significant differences in stride frequency among the different intervention groups at each time point (F = 1.924, *p* = 0.148, partial η^2^ = 0.015) (Table 5).

**Figure 6:**
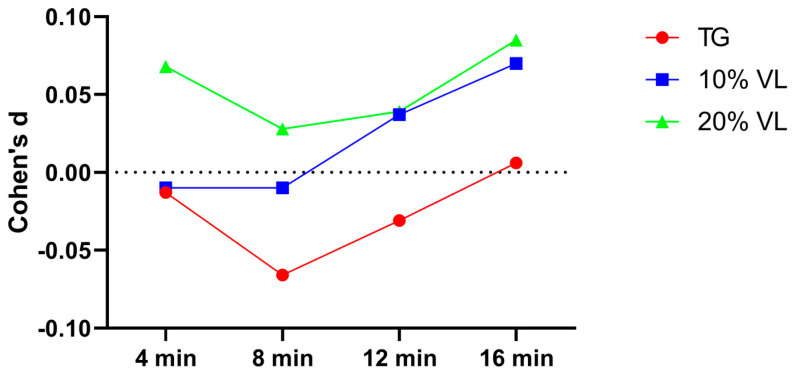
Stride frequency Cohen’s d value of TG, 10% VL, and 20% VL interventions in 0–20 m phase.

Individual effect sizes calculated using Cohen’s d values showed that for the TG group, the effect sizes at each testing point were −0.013, −0.066, −0.031, and 0.006, respectively. For the 10% VL group, the effect sizes were −0.010, −0.010, 0.037, and 0.070, respectively. Notably, the 20% VL group exhibited effect sizes of 0.068, 0.028, 0.039, and 0.085 at each testing point (Figure 6).

## 4. Discussion

This study aims to investigate the effects of different velocity losses on PAPE in sprint athletes. The study primarily found that, at specific time points following interventions with different training conditioning activities, a 20% velocity loss (20% VL) is more effective in inducing PAPE in sprint athletes compared to a 10%VL. This was evidenced by a significant improvement in sprint performance in the 0–20 m phase at the 4 min mark when employing a 20% VL warm-up. Although no significant differences were observed at other time intervals (8, 12, and 16 min), the time taken for both the 0–10 m and 0–20 m sprints was lower when using the 20% VL warm-up compared to the 10% VL and 1RM-based interventions. This finding provides a reference for sprint athletes to adopt velocity-based intervention protocols. Implementing PAPE protocols based on velocity loss can, to some extent, replace traditional 1RM-based PAPE protocols, addressing the limitations of traditional training methods in accurately controlling training loads based on individual states. A 20% VL warm-up may represent the optimal load for inducing PAPE in sprint athletes, enhancing their ability to initiate and accelerate during sprints. Given the lack of significant changes and considering the effect size, we believe the results support the hypothesis that “compared to traditional PAPE protocols based on 1RM, the velocity loss-based (20% VL) PAPE protocol more effectively induces PAPE effects in sprint athletes at the 4-min mark”. However, the results do not support the hypothesis that “stride length and stride frequency are potential factors influencing sprint performance”.

The purpose of PAPE is to optimize an athlete’s physical state before engaging in sports activities, aiming to enhance overall athletic performance. PAPE is commonly believed to improve explosive muscle capabilities, as observed in activities like vertical jumps and sprints, leading to more powerful and rapid muscle contractions, thereby elevating an athlete’s explosive performance [22]. It is generally accepted that the potential effect can be induced, as long as the minimum intensity and sufficient rest time are provided. While the intensity of 65% 1RM is sufficient to elicit a potentiation effect, intensities ranging from 85% to 90% 1RM can yield even greater results [23].

In this study, the use of a 20% VL significantly improved sprint performance in the 0–20 m phase at the 4 min mark. This suggests that performing squats with a 20% VL, followed by sprinting after 4 min, maximizes the PAPE effect. Similar findings have been reported in research involving sprinters, where a study focusing on sprinters revealed that PAPE induced by a 90% 1RM significantly improved arrowhead agility test performance (*p* < 0.001) and repeated sprint ability (*p* = 0.002), with a shorter 20 m sprint time post-PAPE (*p* = 0.005), demonstrating the effectiveness of PAPE for sprint performance [24]. In the context of long-term training, a study based on velocity-based training (VBT) indicated that, despite the 5% VL group having only 32.6% of the repetitions compared to the 20% VL group, after 7 weeks of VBT training, both groups showed similar gains in strength, jumping, and sprinting performance [25].

Following intervention with 20% VL at other time points (8 min, 12 min, and 16 min), there were no significant differences in athletic performance, indicating a time-dependent occurrence of PAPE. A study inducing PAPE through Barbell Hip Thrusts (BHT) found that at 15 s, only an 85% 1RM load induced PAPE, while the 50% 1RM load did not significantly impact performance. However, both PAPE-inducing protocols resulted in significant improvements in 10 m and 15 m sprints at 4 min and 8 min [26]. After a pre-stimulus of 91% 1RM, a notable improvement in sprint performance was observed compared to the baseline, characterized by a best sprint time within 8 min [27].

Additionally, PAPE may also be related to the choice of assessment metrics, as adjustments in rest intervals appear to influence the magnitude of post-activation potentiation and jump height. For instance, in a meta-analysis examining the acute effects of interval timing on jump performance-related PAPE, it was found that the 0–3 min interval had a detrimental impact on jump performance, while the 8–12 min range positively affected jump height [28]. Possible reasons for the poor jumping performance due to the 0–3 min rest interval may include fatigue, inadequate neural adaptation, and insufficient warm-up effects. The combined effect of these factors may prevent the full recovery of the nervous and muscular systems, thereby hindering athletes from performing at their best. Another meta-analysis reported that, for power output, a rest interval of 7–10 min was deemed the most ideal for inducing PAPE effects (ES = 0.70) [29].

In this study, not all load stimuli resulted in performance gains. Specifically, both traditional squat training based on 1RM load intensity and squat training with a 10% VL load intensity were found to be ineffective in enhancing performance in the 0–10 m, 10–20 m, and 0–20 m phases. Although it has been suggested that a load intensity of 65% 1RM can induce a potentiation effect, some studies still report negative outcomes. For instance, Reardon et al. found that moderate and high-intensity conditioning activities (three sets of 10 repetitions or 3 repetitions, at 75% 1RM and 90% 1RM, respectively) did not improve performance scores, despite inducing acute muscle structural changes [30]. Several reasons could explain this phenomenon, such as considerable variability in individual responses to pre-activation stimuli, as well as the nature, intensity, and duration of the stimuli, all of which may influence the occurrence of PAPE. Additionally, the pre-activation state of individuals, including fatigue level, psychological state, and nutritional status, may impact the manifestation of the PAPE effect [3]. In the context of this study, neither the 85% 1RM nor the 10% VL load intensity elicited a PAPE effect. It is plausible to suggest that highly trained athletes, due to their extensive professional training, may have adapted their neuromuscular systems to typical pre-activation stimuli. This adaptation could potentially result in their neural systems being less responsive to stimuli that induce PAPE.

Several factors influence post-activation potentiation (PAPE), including interindividual variability, pre-activation methods, recovery time, and the type of training. From an anatomical and physiological perspective, research suggests that PAPE is linked to increased calcium ion sensitivity in muscle cells, the enhanced recruitment capacity of fast muscle fiber motor units, and alterations in the pennation angle during muscle contraction [31]. Studies indicate that enhancing the sensitivity of myosin and actin-binding sites to calcium ions and increasing the influx of calcium ions into the sarcoplasmic reticulum requires rapid muscle contraction [32]. Pre-activating muscle contraction may increase the release of neurotransmitters, enhance the efficiency of neurotransmitter transmission, or reduce the likelihood of failure in action potential transmission at axon branch points, thereby increasing the recruitment of fast muscle fiber units [16,33]. Additionally, changes in the pennation angle of muscle fibers under 20% VL resistance activation are conducive to transferring force to the relevant tendons, thereby improving the mechanical efficiency of muscle fibers [34]. Santanielo et al. found that the pennation angle increased to some extent after non-exhaustive strength training loads that approximated 20% VL [35].

In the context of a 100 m sprint, both stride length and stride frequency can impact performance. Stride length can be trained through methods such as improving flexibility, enhancing technique, and exerting more force on the ground, while stride frequency training focuses more on the central nervous system’s reaction time [36]. In theory, PAPE may enhance muscle strength and central nervous system reactivity, thus influencing stride length and frequency. Changes in stride length and frequency could ultimately be the reasons behind variations in sprint performance. Therefore, this study attempts to explain the reasons for PAPE from the perspectives of stride length and frequency. Changes in stride length and frequency before and after the intervention were tested, but no significant alterations were observed. Looking at the trend, at the 8 min and 12 min stages, the 20% VL group showed slightly higher stride lengths than the 10% VL group and TG group, while their stride frequency was slightly lower than that of the 10% VL group and TG group. Some studies suggest that in a 100 m sprint, stride length contributes more significantly to speed than stride frequency. Excessive acceleration (correlated with high stride frequency) during rapid strides after the start negatively affects speed throughout the entire 100 m sprint. Therefore, it is necessary to control stride frequency and increase stride length in the initial stages of rapid strides after leaving the starting line, to reduce or eliminate the negative impact of excessive acceleration [37]. This, to some extent, explains why the 20% VL group in this study exhibited a trend of better performance than the 10% VL group and TG group.

Based on the observations from our study, we propose the hypothesis that the conditioning activity performed after reaching the 20% VL protocol may enhance the neuromuscular system’s readiness and force generation capacity, thereby improving subsequent performance. The lack of significant changes in stride length and frequency may be due to this neuromuscular adaptation, which primarily affects force exertion rather than movement mechanics. Future research should further explore this hypothesis, investigating the specific neuromuscular mechanisms involved in different VL protocols and their relationships.

### Limitations of the Study

This study introduces novel strategies aimed at enhancing sprinters’ starting ability for both competitive events and training sessions. Nevertheless, there are several limitations to consider in this research: ① The study solely examined two load intensities (10% VL and 20% VL). In reality, incremental increases of 5% VL might yield varying PAPE effects. Future investigations should incorporate a more diverse range of load intensities to comprehensively explore VL’s impact on PAPE. ② Evaluation metrics in this study focused exclusively on 20 m sprint performance, potentially neglecting VL’s PAPE effects on other parameters such as vertical jump, 100 m sprint time, and power output. ③ While this study concentrated on stride length and frequency as factors influencing PAPE, muscle neuromuscular function and fatigue status could be more accurately reflected through electromyography (EMG) signals [38]. Therefore, future studies could delve into surface EMG signals for a more nuanced analysis. ④ A limitation of this study is its relatively small sample size, potentially constraining the generalizability of findings to broader populations. Enlarging the sample size would bolster the statistical power and reliability of the results. ⑤ Subjective perception of intensity (RPE) during training was not recorded or reported, which could offer supplementary information for controlling and comparing the effects of different intervention protocols. ⑥ Although the equipment used underwent validation in previous studies and demonstrated high reliability, an independent assessment of its validity and reliability was not conducted. ⑦ This study compared the effects of three training protocols on sprinters’ PAPE. Future research should consider implementing these protocols on different days within the same participant group. This adjustment would help mitigate individual differences and foster a clearer comprehension of each protocol’s impact on sprint performance.

## 5. Conclusions

In conclusion, the velocity loss-based PAPE protocol (20% VL) demonstrated a superior induction of PAPE effects in sprint athletes at the 4 min mark compared to traditional 1RM-based PAPE protocols. However, no significant differences were observed between the two protocols at the 8, 12, and 16 min marks. This suggests that in practical application, sprint athletes may adopt a 20% VL load intensity with a 4 min rest interval to achieve optimal PAPE effects.

## Figures and Tables

**Table 1:** The means and confidence intervals of different time points for each group in the 0–10 m phase.

	Pre		4 min		8 min		12 min		16 min		Group	Time	Group × Time
	mean ± SD	95% CI	mean ± SD	95% CI	mean ± SD	95% CI	mean ± SD	95% CI	mean ± SD	95% CI	*p*	*p*	*p*
TG	1.681 ± 0.017	1.647~1.714	1.685 ± 0.016	1.654~1.717	1.664 ± 0.017	1.629~1.699	1.683 ± 0.017	1.648~1.718	1.686 ± 0.017	1.652~1.72	0.169	0.247	0.083
10% VL	1.691 ± 0.017	1.656~1.725	1.71 ± 0.016	1.678~1.742	1.688 ± 0.018	1.653~1.724	1.697 ± 0.018	1.662~1.733	1.694 ± 0.017	1.659~1.729			
20% VL	1.69 ± 0.017	1.658~1.723	1.657 ± 0.015	1.626~1.687	1.656 ± 0.017	1.622~1.691	1.672 ± 0.017	1.639~1.706	1.665 ± 0.017	1.632~1.698			

**Table 2:** The means and confidence intervals of different time points for each group in the 10–20 m phase.

	Pre		4 min		8 min		12 min		16 min		Group	Time	Group × Time
	Mean ± SD	95% CI	Mean ± SD	95% CI	Mean ± SD	95% CI	Mean ± SD	95% CI	Mean ± SD	95% CI	*p*	*p*	*p*
TG	1.202 ± 0.009	1.184~1.22	1.213 ± 0.009	1.196~1.23	1.225 ± 0.014	1.197~1.254	1.206 ± 0.01	1.187~1.225	1.2 ± 0.011	1.178~1.223	0.481	0.237	0.493
10% VL	1.209 ± 0.009	1.191~1.227	1.199 ± 0.009	1.182~1.216	1.203 ± 0.014	1.175~1.232	1.203 ± 0.01	1.184~1.222	1.188 ± 0.011	1.166~1.21			
20% VL	1.199 ± 0.009	1.18~1.217	1.193 ± 0.009	1.175~1.21	1.199 ± 0.014	1.17~1.227	1.195 ± 0.01	1.176~1.214	1.195 ± 0.011	1.173~1.217			

**Table 3:** The means and confidence intervals of different time points for each group in the 0–20 m phase.

	Pre		4 min		8 min		12 min		16 min		Group	Time	Group × Time
	Mean ± SD	95% CI	Mean ± SD	95% CI	Mean ± SD	*p*	*p*	*p*	Mean ± SD	95% CI	*p*	*p*	*p*
TG	2.883 ± 0.021	2.842~2.924	2.896 ± 0.021	2.854~2.939	2.885 ± 0.022	2.841~2.93	2.887 ± 0.022	2.842~2.932	2.887 ± 0.024	2.84~2.935	0.208	0.509	0.037
10% VL	2.898 ± 0.021	2.857~2.939	2.904 ± 0.021	2.861~2.947	2.891 ± 0.022	2.847~2.936	2.89 ± 0.022	2.845~2.934	2.878 ± 0.024	2.831~2.926			
20% VL	2.895 ± 0.02	2.855~2.936	2.857 ± 0.021	2.815~2.899	2.86 ± 0.022	2.817~2.904	2.872 ± 0.022	2.828~2.916	2.866 ± 0.023	2.819~2.912			

**Table 4:** Stride length mean and confidence interval for each group at different time points in 0–20 m phase.

	Pre		4 min		8 min		12 min		16 min		Group	Time	Group × Time
	Mean ± SD	95% CI	Mean ± SD	95% CI	Mean ± SD	95% CI	Mean ± SD	95% CI	Mean ± SD	95% CI	*p*	*p*	*p*
TG	337.797 ± 5.229	327.503~348.091	334.942 ± 5.321	324.468~345.416	338.634 ± 5.573	327.663~349.605	335.569 ± 5.337	325.062~346.075	337.19 ± 5.221	326.912~347.469	0.159	0.061	0.388
10% VL	334.472 ± 5.119	324.395~344.549	337.085 ± 5.209	326.831~347.339	337.356 ± 5.456	326.616~348.096	338.342 ± 5.225	328.057~348.627	336.573 ± 5.111	326.511~346.635			
20% VL	335.312 ± 5.229	325.018~345.606	337.466 ± 5.321	326.991~347.94	341.188 ± 5.573	330.217~352.16	341.672 ± 5.337	331.166~352.178	338.498 ± 5.221	328.22~348.777			

**Table 5:** Stride frequency mean and confidence interval for each group at different time points in 0–20 m phase.

	Pre		4 min		8 min		12 min		16 min		Group	Time	Group × Time
	Mean ± SD	95% CI	Mean ± SD	95% CI	Mean ± SD	95% CI	Mean ± SD	95% CI	Mean ± SD	95% CI	*p*	*p*	*p*
TG	4.37 ± 1.682	1.059~7.681	4.418 ± 1.686	1.099~7.737	4.411 ± 1.834	0.8~8.021	4.434 ± 1.746	0.996~7.872	4.387 ± 1.848	0.749~8.026	0.148	0.645	0.924
10% VL	7.293 ± 1.664	4.017~10.568	7.3 ± 1.668	4.017~10.584	7.551 ± 1.814	3.98~11.123	7.408 ± 1.728	4.007~10.809	7.605 ± 1.829	4.005~11.204			
20% VL	4.437 ± 1.682	1.126~7.747	4.425 ± 1.686	1.106~7.743	4.39 ± 1.834	0.78~8.001	4.398 ± 1.746	0.961~7.836	4.422 ± 1.848	0.784~8.061			

## Data Availability

The authors declare that the dataset is available upon request.
